# Bis(1,10-phenanthroline-κ^2^ *N*,*N*′)(sulfato-*O*)copper(II) ethane-1,2-diol monosolvate

**DOI:** 10.1107/S1600536811031175

**Published:** 2011-08-11

**Authors:** Kai-Long Zhong

**Affiliations:** a Department of Applied Chemistry, Nanjing College of Chemical Technology, Nanjing, 210048, People’s Republic of China

## Abstract

In the title compound, [Cu(SO_4_)(C_12_H_8_N_2_)_2_]·C_2_H_6_O_2_, the Cu^II^ ion is five-coordinated in a distorted square-pyramidal manner by four N atoms from two chelating 1,10-phenanthroline (phen) ligands and one O atom from a monodentate sulfate anion. The four N atoms comprise a square and the one O atom the apex of a square pyramid. The two chelating N_2_C_2_ groups are oriented at 71.1 (2)°. In the crystal, the components are connected by inter­molecular O—H⋯O hydrogen bonding. The presence of pseudosymmetry in the structure suggests the higher symmetry space group *C*2/*c*, but attempts to refine the structure in this space group resulted in an unsatisfactory model.

## Related literature

For the propane-1,2-diol solvate of the title complex, see: Zhong (2011 ▶). For related structures of transition metal complexes of phen and for background references, see: Zhong *et al.* (2006 ▶; 2009 ▶); Zhong & Cui (2010 ▶); Ni *et al.* (2010 ▶); Zhong (2010 ▶); Cui *et al.* (2010 ▶).
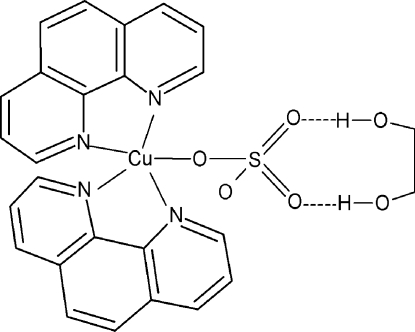


## Experimental

### 

#### Crystal data

[Cu(SO_4_)(C_12_H_8_N_2_)_2_]·C_2_H_6_O_2_
*M* *_r_* = 582.08
Monoclinic, 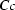

*a* = 17.666 (4) Å
*b* = 11.992 (2) Å
*c* = 13.122 (3) Å
β = 120.96 (3)°
*V* = 2383.8 (11) Å^3^
*Z* = 4
Mo *K*α radiation
μ = 1.06 mm^−1^
*T* = 223 K
0.40 × 0.35 × 0.25 mm

#### Data collection

Rigaku Mercury CCD diffractometer
Absorption correction: multi-scan (*REQAB*; Jacobson, 1998 ▶) *T*_min_ = 0.776, *T*_max_ = 1.000
6663 measured reflections
4089 independent reflections
3835 reflections with *I* > 2/s(*I*)
*R*_int_ = 0.018

#### Refinement

*R*[*F* ^2^ > 2σ(*F* ^2^)] = 0.039
*wR*(*F* ^2^) = 0.102
*S* = 1.06
4089 reflections
344 parameters
2 restraints
H-atom parameters constrained
Δρ_max_ = 1.07 e Å^−3^
Δρ_min_ = −0.81 e Å^−3^
Absolute structure: Flack (1983 ▶), 1396 Friedel pairs
Flack parameter: 0.254 (14)

### 

Data collection: *CrystalClear* (Rigaku, 2007 ▶); cell refinement: *CrystalClear*; data reduction: *CrystalClear*; program(s) used to solve structure: *SHELXS97* (Sheldrick, 2008 ▶); program(s) used to refine structure: *SHELXL97* (Sheldrick, 2008 ▶); molecular graphics: *XP* in *SHELXTL* (Sheldrick, 2008 ▶); software used to prepare material for publication: *SHELXTL*.

## Supplementary Material

Crystal structure: contains datablock(s) global, I. DOI: 10.1107/S1600536811031175/bt5595sup1.cif

Structure factors: contains datablock(s) I. DOI: 10.1107/S1600536811031175/bt5595Isup2.hkl

Additional supplementary materials:  crystallographic information; 3D view; checkCIF report

## Figures and Tables

**Table 1:** Selected geometric parameters (Å, °)

Cu1—O1	1.957 (3)
Cu1—N2	1.990 (4)
Cu1—N3	2.006 (4)
Cu1—N4	2.073 (4)
Cu1—N1	2.162 (4)
S1—O4	1.469 (3)
S1—O2	1.470 (3)
S1—O3	1.475 (3)
S1—O1	1.504 (3)

**Table 2:** Hydrogen-bond geometry (Å, °)

*D*—H⋯*A*	*D*—H	H⋯*A*	*D*⋯*A*	*D*—H⋯*A*
O6—H6*B*⋯O4	0.82	1.93	2.751 (5)	176
O5—H5*B*⋯O3	0.82	1.98	2.798 (5)	171

**Fig. 1.**
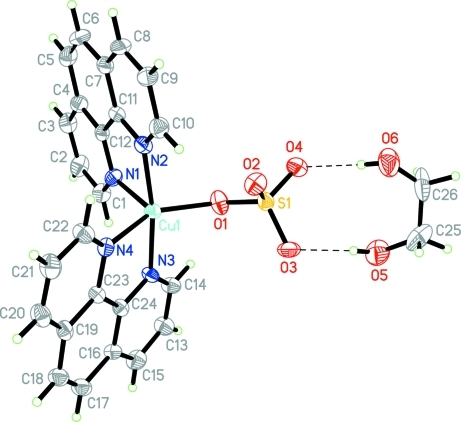
The molecular structure showing the atom-numbering scheme and with displacement ellipsoids drawn at the 50% probability level. The dashed lines represent O—H···O interactions.

## supplementary crystallographic information

### Comment

In the past few years, we have reported many metal-Phen complexes with bidentate-chelating sulfate auxiliary ligand *via* a alcohlol-solvothermal reaction, such as [CoSO_4_(C_12_H_8_N_2_)_2_].C_2_H_6_O_2_ (Zhong *et al.*, 2006), [NiSO_4_(C_12_H_8_N_2_)_2_].C_2_H_6_O_2_ (Zhong *et al.*, 2009), [CoSO_4_(C_12_H_8_N_2_)_2_].C_3_H_8_O_2_ (Zhong, 2010), [ZnSO_4_(C_12_H_8_N_2_)_2_].C_3_H_8_O_2_ (Cui *et al.*, 2010), [NiSO_4_(C_12_H_8_N_2_)_2_].C_3_H_8_O_2_ (Ni *et al.*, 2010 and [CdSO_4_(C_12_H_8_N_2_)_2_].C_3_H_8_O_2_ (Zhong & Cui, 2010). The title compound was obtained during an attempt to synthesize a complex of Cu-complex with bidentate-chelating sulfate ligand by the similar route. The crystal structure of the title complex [CuSO_4_(C_12_H_8_N_2_)_2_].C_2_H_6_O_2_, (I) has not hitherto been reported.

Single crystal X-ray diffraction experiment indicated that the title compound is isostructual to the recently reported Cu(C_12_H_8_N_2_)_2_(SO_4_).C_3_H_8_O_2_, (II) (Zhong, 2011), the Cu^II^ metal ion is five-coordinated in a distorted square-pyramidal coordination environment formed by four N atoms(N1, N2, N3 and N4) from two chelating phen ligands and an O atoms(O1) from a monodentate sulfate ligand, the N1, N2, N3 and N4 atoms comprise a square, and the O3 atom the apex of a square pyramid surrounding each metal atom. The geometry of the phen and sulfate ligands is in good agreement with those reported in the isomorphs complex(II). The dihedral angle between the two chelating N2C2 groups is 71.1 (2)°, this is smaller than that found in (II) [84.9 (4)°]. The Cu—O bond distance [1.957 (3) Å], the Cu—N bond distance [1.990 (4) - 2.162 (4) Å], and the N—Cu—N bite angle [80.4 (2) - 81.4 (2)°] are in good agreement with observed in (II), [1.901 (1) Å, 1.991 (7) - 2.154 (8)Å and 80.4 (3) - 80.7 (3)°, respectively](see Table 1). The metal complex and solvent entities of (I) are held together by a pair of intermolecular O—H···O hydrogen bonds with the uncoordinated O atoms of the sulfate group(see Table 2 & Fig. 1).

### Experimental

0.2 mmol phen, 0.1 mmol CuSO_4_.5H_2_O, 2.0 ml e thane-1,2-diol and 1.0 ml water were mixed and placed in a thick Pyrex tube, which was sealed and heated to 453 K for 96 h, whereupon Blue block-shaped crystals of (I) were obtained. The presence of pseudo-symmetry in the structure suggests a higher symmetry space group *C*2/*c*. But attempts to refine the structure in the space group *C*2/*c* resulted in a disorder model with high *R* and *wR* values. Hence the requirement to solve in *Cc*. The reported Flack parameter was refined as s full least-squares and obtained by TWIN/BASF procedure in *SHELXL* (Sheldrick, 2008).

### Refinement

All H atoms   were positioned geometrically and allowed to ride on their parent atoms, with C—H = 0.93 Å and *U*_iso_(H) = 1.2*U*_eq_(C) or C—H = 0.97 Å and O—H = 0.82 Å; *U*_iso_(H) = 1.2*U*_eq_(C) and 1.5*U*_eq_(O).

### Crystal data

**Table:**

[Cu(SO_4_)(C_12_H_8_N_2_)_2_]·C_2_H_6_O_2_	*F*(000) = 1196
*M**_r_* = 582.08	*D*_x_ = 1.622 Mg m^−^^3^
Monoclinic, *C**c*	Mo *K*α radiation, λ = 0.71073 Å
Hall symbol: C -2yc	Cell parameters from 5713 reflections
*a* = 17.666 (4) Å	θ = 3.2–27.5°
*b* = 11.992 (2) Å	µ = 1.06 mm^−^^1^
*c* = 13.122 (3) Å	*T* = 223 K
β = 120.96 (3)°	Block, green
*V* = 2383.8 (11) Å^3^	0.40 × 0.35 × 0.25 mm
*Z* = 4	

### Data collection

**Table:**

Rigaku Mercury CCD diffractometer	4089 independent reflections
Radiation source: fine-focus sealed tube	3835 reflections with *I* > 2/s(*I*)
Graphite Monochromator	*R*_int_ = 0.018
Detector resolution: 28.5714 pixels mm^-1^	θ_max_ = 27.5°, θ_min_ = 3.2°
ω scans	*h* = −21→22
Absorption correction: multi-scan (REQAB; Jacobson, 1998)	*k* = −13→15
*T*_min_ = 0.776, *T*_max_ = 1.000	*l* = −15→17
6663 measured reflections	

### Refinement

**Table:**

Refinement on *F*^2^	Secondary atom site location: difference Fourier map
Least-squares matrix: full	Hydrogen site location: inferred from neighbouring sites
*R*[*F*^2^ > 2σ(*F*^2^)] = 0.039	H-atom parameters constrained
*wR*(*F*^2^) = 0.102	*w* = 1/[σ^2^(*F*_o_^2^) + (0.064*P*)^2^ + 1.9064*P*] where *P* = (*F*_o_^2^ + 2*F*_c_^2^)/3
*S* = 1.06	(Δ/σ)_max_ < 0.001
4089 reflections	Δρ_max_ = 1.07 e Å^−^^3^
344 parameters	Δρ_min_ = −0.81 e Å^−^^3^
2 restraints	Absolute structure: Flack (1983), 1396 Friedel pairs
Primary atom site location: structure-invariant direct methods	Flack parameter: 0.254 (14)

### Special details

**Table:**

Geometry. All e.s.d.'s (except the e.s.d. in the dihedral angle between two l.s. planes) are estimated using the full covariance matrix. The cell e.s.d.'s are taken into account individually in the estimation of e.s.d.'s in distances, angles and torsion angles; correlations between e.s.d.'s in cell parameters are only used when they are defined by crystal symmetry. An approximate (isotropic) treatment of cell e.s.d.'s is used for estimating e.s.d.'s involving l.s. planes.
Refinement. Refinement of *F*^2^ against ALL reflections. The weighted *R*-factor *wR* and goodness of fit *S* are based on *F*^2^, conventional *R*-factors *R* are based on *F*, with *F* set to zero for negative *F*^2^. The threshold expression of *F*^2^ > σ(*F*^2^) is used only for calculating *R*-factors(gt) *etc*. and is not relevant to the choice of reflections for refinement. *R*-factors based on *F*^2^ are statistically about twice as large as those based on *F*, and *R*- factors based on ALL data will be even larger.

### Fractional atomic coordinates and isotropic or equivalent isotropic displacement parameters (Å^2^)

**Table:**

	*x*	*y*	*z*	*U*_iso_*/*U*_eq_	
Cu1	0.17114 (3)	0.28325 (3)	0.03203 (4)	0.02354 (12)	
S1	0.16061 (6)	0.54366 (7)	0.04366 (9)	0.0262 (2)	
N3	0.2587 (3)	0.2724 (3)	−0.0216 (4)	0.0220 (8)	
N2	0.0827 (3)	0.2673 (3)	0.0820 (4)	0.0253 (8)	
C24	0.3290 (3)	0.2059 (3)	0.0478 (4)	0.0215 (9)	
N1	0.0735 (2)	0.1831 (3)	−0.1135 (3)	0.0246 (8)	
N4	0.2650 (2)	0.1851 (3)	0.1690 (3)	0.0223 (7)	
C11	0.0105 (3)	0.2038 (3)	0.0078 (4)	0.0235 (9)	
C8	−0.0484 (3)	0.2248 (4)	0.1356 (5)	0.0298 (11)	
H8A	−0.0915	0.2107	0.1546	0.036*	
C15	0.3855 (4)	0.2311 (4)	−0.0817 (5)	0.0335 (11)	
H15A	0.4279	0.2186	−0.1023	0.040*	
O4	0.0858 (2)	0.6189 (3)	−0.0263 (3)	0.0336 (7)	
C14	0.2517 (3)	0.3163 (4)	−0.1194 (4)	0.0268 (9)	
H14A	0.2038	0.3622	−0.1669	0.032*	
C10	0.0876 (3)	0.3084 (4)	0.1789 (5)	0.0313 (10)	
H10A	0.1356	0.3532	0.2291	0.038*	
C12	0.0056 (3)	0.1594 (4)	−0.0968 (4)	0.0218 (8)	
C19	0.4077 (3)	0.0962 (3)	0.2325 (4)	0.0255 (10)	
C4	−0.0682 (3)	0.0986 (4)	−0.1775 (4)	0.0264 (10)	
O3	0.2432 (2)	0.5960 (3)	0.0654 (3)	0.0365 (8)	
C13	0.3122 (4)	0.2966 (4)	−0.1525 (5)	0.0320 (11)	
H13A	0.3042	0.3272	−0.2225	0.038*	
C9	0.0224 (4)	0.2870 (3)	0.2096 (5)	0.0298 (11)	
H9A	0.0287	0.3154	0.2795	0.036*	
C18	0.4738 (3)	0.0761 (4)	0.2050 (5)	0.0302 (11)	
H18A	0.5224	0.0334	0.2573	0.036*	
C5	−0.1378 (3)	0.0776 (4)	−0.1545 (4)	0.0316 (11)	
H5A	−0.1872	0.0370	−0.2087	0.038*	
C7	−0.0582 (3)	0.1810 (4)	0.0300 (4)	0.0251 (9)	
C16	0.3945 (3)	0.1835 (4)	0.0227 (4)	0.0260 (9)	
C17	0.4695 (3)	0.1164 (4)	0.1054 (4)	0.0315 (10)	
H17A	0.5145	0.1010	0.0904	0.038*	
C3	−0.0707 (3)	0.0591 (4)	−0.2814 (4)	0.0310 (10)	
H3A	−0.1183	0.0171	−0.3374	0.037*	
C23	0.3340 (3)	0.1616 (4)	0.1529 (4)	0.0218 (8)	
C6	−0.1316 (3)	0.1171 (4)	−0.0534 (4)	0.0318 (10)	
H6A	−0.1766	0.1018	−0.0385	0.038*	
C20	0.4089 (3)	0.0575 (4)	0.3340 (4)	0.0318 (10)	
H20A	0.4558	0.0141	0.3892	0.038*	
C2	−0.0025 (4)	0.0838 (4)	−0.2974 (5)	0.0350 (12)	
H2A	−0.0038	0.0601	−0.3658	0.042*	
C22	0.2710 (3)	0.1466 (4)	0.2686 (4)	0.0294 (10)	
H22A	0.2257	0.1632	0.2826	0.035*	
C21	0.3416 (4)	0.0832 (4)	0.3522 (4)	0.0348 (12)	
H21A	0.3430	0.0584	0.4203	0.042*	
C1	0.0701 (3)	0.1451 (4)	−0.2109 (4)	0.0300 (9)	
H1A	0.1171	0.1593	−0.2220	0.036*	
O1	0.1418 (2)	0.4369 (2)	−0.0255 (3)	0.0357 (7)	
O6	0.1139 (3)	0.8222 (3)	0.0846 (4)	0.0541 (11)	
H6B	0.1059	0.7631	0.0492	0.081*	
O5	0.2232 (3)	0.8087 (3)	−0.0330 (4)	0.0503 (10)	
H5B	0.2288	0.7496	0.0017	0.075*	
C26	0.1316 (6)	0.9055 (4)	0.0264 (7)	0.058 (2)	
H26A	0.1279	0.9770	0.0584	0.070*	
H26B	0.0857	0.9042	−0.0569	0.070*	
C25	0.2177 (4)	0.8984 (5)	0.0348 (6)	0.0586 (18)	
H25A	0.2289	0.9681	0.0072	0.070*	
H25B	0.2635	0.8890	0.1175	0.070*	
O2	0.1672 (2)	0.5194 (2)	0.1578 (2)	0.0332 (6)	

### Atomic displacement parameters (Å^2^)

**Table:**

	*U*^11^	*U*^22^	*U*^33^	*U*^12^	*U*^13^	*U*^23^
Cu1	0.0233 (2)	0.0247 (2)	0.0272 (2)	0.0012 (2)	0.01621 (16)	0.0011 (3)
S1	0.0221 (5)	0.0222 (4)	0.0277 (5)	0.0012 (4)	0.0079 (4)	−0.0003 (4)
N3	0.022 (2)	0.0252 (19)	0.0229 (19)	0.0066 (14)	0.0142 (17)	0.0048 (15)
N2	0.025 (2)	0.0251 (19)	0.027 (2)	0.0026 (15)	0.0137 (19)	−0.0009 (16)
C24	0.024 (2)	0.021 (2)	0.019 (2)	0.0000 (16)	0.0106 (18)	−0.0046 (16)
N1	0.0242 (19)	0.0254 (19)	0.0228 (18)	−0.0047 (15)	0.0110 (16)	−0.0030 (15)
N4	0.0291 (19)	0.0201 (17)	0.0215 (17)	−0.0015 (15)	0.0158 (16)	−0.0010 (15)
C11	0.020 (2)	0.022 (2)	0.028 (2)	0.0029 (16)	0.012 (2)	0.0011 (18)
C8	0.027 (3)	0.033 (3)	0.038 (3)	−0.0001 (19)	0.023 (2)	0.005 (2)
C15	0.040 (3)	0.037 (3)	0.034 (3)	−0.003 (2)	0.026 (2)	−0.001 (2)
O4	0.0286 (16)	0.0358 (16)	0.0351 (16)	0.0043 (13)	0.0155 (14)	0.0010 (14)
C14	0.025 (2)	0.035 (2)	0.023 (2)	0.0033 (19)	0.015 (2)	0.005 (2)
C10	0.035 (3)	0.028 (2)	0.034 (3)	−0.002 (2)	0.019 (2)	−0.003 (2)
C12	0.024 (2)	0.0184 (18)	0.0227 (19)	0.0034 (17)	0.0119 (18)	0.0053 (17)
C19	0.025 (2)	0.018 (2)	0.023 (2)	−0.0011 (17)	0.0053 (19)	−0.0057 (18)
C4	0.024 (2)	0.028 (2)	0.025 (2)	0.0002 (18)	0.010 (2)	0.0010 (19)
O3	0.0219 (16)	0.0410 (19)	0.049 (2)	0.0042 (13)	0.0197 (15)	0.0121 (15)
C13	0.034 (3)	0.038 (3)	0.029 (3)	0.004 (2)	0.019 (2)	0.005 (2)
C9	0.037 (3)	0.031 (3)	0.033 (3)	0.0004 (19)	0.026 (2)	0.0018 (19)
C18	0.022 (2)	0.032 (3)	0.032 (2)	0.0006 (18)	0.010 (2)	−0.002 (2)
C5	0.025 (3)	0.028 (2)	0.030 (2)	−0.0058 (18)	0.005 (2)	−0.0016 (19)
C7	0.027 (2)	0.024 (2)	0.028 (2)	0.0057 (18)	0.016 (2)	0.0078 (19)
C16	0.020 (2)	0.027 (2)	0.031 (2)	−0.0002 (18)	0.013 (2)	−0.001 (2)
C17	0.023 (2)	0.032 (2)	0.035 (2)	−0.0005 (19)	0.012 (2)	−0.006 (2)
C3	0.033 (3)	0.029 (2)	0.025 (2)	−0.001 (2)	0.010 (2)	−0.004 (2)
C23	0.021 (2)	0.022 (2)	0.0214 (19)	0.0024 (17)	0.0105 (18)	0.0008 (17)
C6	0.025 (2)	0.037 (3)	0.038 (3)	−0.006 (2)	0.019 (2)	0.001 (2)
C20	0.031 (3)	0.030 (3)	0.025 (2)	0.006 (2)	0.007 (2)	0.002 (2)
C2	0.046 (3)	0.032 (3)	0.028 (2)	−0.007 (2)	0.020 (2)	−0.006 (2)
C22	0.033 (3)	0.035 (2)	0.027 (2)	0.008 (2)	0.021 (2)	0.006 (2)
C21	0.040 (3)	0.035 (3)	0.025 (2)	0.004 (2)	0.014 (2)	0.009 (2)
C1	0.035 (3)	0.030 (2)	0.026 (2)	−0.001 (2)	0.017 (2)	−0.002 (2)
O1	0.0474 (18)	0.0267 (13)	0.0325 (15)	0.0058 (13)	0.0201 (14)	−0.0012 (12)
O6	0.084 (3)	0.037 (2)	0.069 (3)	−0.003 (2)	0.059 (3)	−0.008 (2)
O5	0.068 (3)	0.047 (2)	0.057 (2)	−0.006 (2)	0.048 (2)	0.0026 (19)
C26	0.111 (6)	0.020 (2)	0.068 (4)	−0.002 (3)	0.065 (5)	−0.006 (3)
C25	0.053 (4)	0.059 (4)	0.058 (4)	−0.029 (3)	0.025 (3)	−0.014 (3)
O2	0.0381 (17)	0.0364 (15)	0.0265 (13)	−0.0035 (13)	0.0176 (13)	0.0002 (13)

### Geometric parameters (Å, °)

**Table:**

Cu1—O1	1.957 (3)	C19—C18	1.409 (7)
Cu1—N2	1.990 (4)	C19—C23	1.415 (6)
Cu1—N3	2.006 (4)	C4—C3	1.422 (7)
Cu1—N4	2.073 (4)	C4—C5	1.432 (7)
Cu1—N1	2.162 (4)	C13—H13A	0.9300
S1—O4	1.469 (3)	C9—H9A	0.9300
S1—O2	1.470 (3)	C18—C17	1.358 (7)
S1—O3	1.475 (3)	C18—H18A	0.9300
S1—O1	1.504 (3)	C5—C6	1.359 (7)
N3—C14	1.333 (6)	C5—H5A	0.9300
N3—C24	1.358 (6)	C7—C6	1.416 (7)
N2—C10	1.324 (7)	C16—C17	1.451 (7)
N2—C11	1.371 (6)	C17—H17A	0.9300
C24—C16	1.382 (6)	C3—C2	1.358 (7)
C24—C23	1.437 (6)	C3—H3A	0.9300
N1—C1	1.330 (6)	C6—H6A	0.9300
N1—C12	1.354 (5)	C20—C21	1.363 (7)
N4—C22	1.338 (6)	C20—H20A	0.9300
N4—C23	1.369 (5)	C2—C1	1.406 (7)
C11—C7	1.412 (6)	C2—H2A	0.9300
C11—C12	1.433 (6)	C22—C21	1.389 (7)
C8—C9	1.347 (8)	C22—H22A	0.9300
C8—C7	1.408 (7)	C21—H21A	0.9300
C8—H8A	0.9300	C1—H1A	0.9300
C15—C13	1.386 (8)	O6—C26	1.386 (7)
C15—C16	1.415 (7)	O6—H6B	0.8200
C15—H15A	0.9300	O5—C25	1.430 (7)
C14—C13	1.365 (7)	O5—H5B	0.8200
C14—H14A	0.9300	C26—C25	1.472 (8)
C10—C9	1.426 (7)	C26—H26A	0.9700
C10—H10A	0.9300	C26—H26B	0.9700
C12—C4	1.390 (6)	C25—H25A	0.9700
C19—C20	1.401 (7)	C25—H25B	0.9700
			
O1—Cu1—N2	96.90 (14)	C8—C9—C10	118.8 (5)
O1—Cu1—N3	91.43 (13)	C8—C9—H9A	120.6
N2—Cu1—N3	170.72 (10)	C10—C9—H9A	120.6
O1—Cu1—N4	143.49 (13)	C17—C18—C19	122.8 (5)
N2—Cu1—N4	94.19 (15)	C17—C18—H18A	118.6
N3—Cu1—N4	81.39 (15)	C19—C18—H18A	118.6
O1—Cu1—N1	104.46 (13)	C6—C5—C4	119.8 (4)
N2—Cu1—N1	80.37 (15)	C6—C5—H5A	120.1
N3—Cu1—N1	93.63 (15)	C4—C5—H5A	120.1
N4—Cu1—N1	111.67 (10)	C8—C7—C11	116.6 (4)
O4—S1—O2	109.46 (18)	C8—C7—C6	124.2 (4)
O4—S1—O3	110.01 (16)	C11—C7—C6	119.2 (4)
O2—S1—O3	109.6 (2)	C24—C16—C15	117.5 (4)
O4—S1—O1	107.30 (19)	C24—C16—C17	119.0 (4)
O2—S1—O1	108.83 (17)	C15—C16—C17	123.5 (4)
O3—S1—O1	111.61 (19)	C18—C17—C16	119.6 (5)
C14—N3—C24	118.5 (4)	C18—C17—H17A	120.2
C14—N3—Cu1	127.3 (3)	C16—C17—H17A	120.2
C24—N3—Cu1	114.0 (3)	C2—C3—C4	118.9 (4)
C10—N2—C11	117.8 (4)	C2—C3—H3A	120.6
C10—N2—Cu1	127.3 (4)	C4—C3—H3A	120.6
C11—N2—Cu1	114.9 (3)	N4—C23—C19	123.7 (4)
N3—C24—C16	122.9 (4)	N4—C23—C24	116.9 (4)
N3—C24—C23	116.3 (4)	C19—C23—C24	119.4 (4)
C16—C24—C23	120.7 (4)	C5—C6—C7	121.7 (4)
C1—N1—C12	118.5 (4)	C5—C6—H6A	119.2
C1—N1—Cu1	131.3 (3)	C7—C6—H6A	119.2
C12—N1—Cu1	110.2 (3)	C21—C20—C19	120.3 (4)
C22—N4—C23	116.7 (4)	C21—C20—H20A	119.9
C22—N4—Cu1	132.0 (3)	C19—C20—H20A	119.9
C23—N4—Cu1	111.2 (3)	C3—C2—C1	120.1 (5)
N2—C11—C7	123.1 (4)	C3—C2—H2A	119.9
N2—C11—C12	117.5 (4)	C1—C2—H2A	119.9
C7—C11—C12	119.4 (4)	N4—C22—C21	123.3 (4)
C9—C8—C7	121.0 (5)	N4—C22—H22A	118.4
C9—C8—H8A	119.5	C21—C22—H22A	118.4
C7—C8—H8A	119.5	C20—C21—C22	119.6 (4)
C13—C15—C16	118.6 (5)	C20—C21—H21A	120.2
C13—C15—H15A	120.7	C22—C21—H21A	120.2
C16—C15—H15A	120.7	N1—C1—C2	121.8 (4)
N3—C14—C13	122.6 (4)	N1—C1—H1A	119.1
N3—C14—H14A	118.7	C2—C1—H1A	119.1
C13—C14—H14A	118.7	S1—O1—Cu1	129.51 (18)
N2—C10—C9	122.7 (5)	C26—O6—H6B	109.5
N2—C10—H10A	118.6	C25—O5—H5B	109.5
C9—C10—H10A	118.6	O6—C26—C25	115.7 (6)
N1—C12—C4	123.3 (4)	O6—C26—H26A	108.3
N1—C12—C11	117.1 (4)	C25—C26—H26A	108.3
C4—C12—C11	119.7 (4)	O6—C26—H26B	108.3
C20—C19—C18	125.2 (4)	C25—C26—H26B	108.3
C20—C19—C23	116.4 (4)	H26A—C26—H26B	107.4
C18—C19—C23	118.4 (4)	O5—C25—C26	113.3 (5)
C12—C4—C3	117.4 (4)	O5—C25—H25A	108.9
C12—C4—C5	120.2 (4)	C26—C25—H25A	108.9
C3—C4—C5	122.4 (4)	O5—C25—H25B	108.9
C14—C13—C15	119.9 (5)	C26—C25—H25B	108.9
C14—C13—H13A	120.0	H25A—C25—H25B	107.7
C15—C13—H13A	120.0		
			
O1—Cu1—N3—C14	−37.2 (4)	N2—C10—C9—C8	−1.8 (7)
N4—Cu1—N3—C14	178.8 (4)	C20—C19—C18—C17	−179.6 (5)
N1—Cu1—N3—C14	67.4 (4)	C23—C19—C18—C17	−0.2 (7)
O1—Cu1—N3—C24	147.5 (3)	C12—C4—C5—C6	−0.1 (7)
N4—Cu1—N3—C24	3.5 (3)	C3—C4—C5—C6	179.7 (5)
N1—Cu1—N3—C24	−107.9 (3)	C9—C8—C7—C11	1.2 (7)
O1—Cu1—N2—C10	−76.9 (4)	C9—C8—C7—C6	−178.6 (4)
N4—Cu1—N2—C10	68.3 (4)	N2—C11—C7—C8	−1.6 (6)
N1—Cu1—N2—C10	179.6 (4)	C12—C11—C7—C8	178.5 (4)
O1—Cu1—N2—C11	105.0 (3)	N2—C11—C7—C6	178.2 (4)
N4—Cu1—N2—C11	−109.8 (3)	C12—C11—C7—C6	−1.6 (6)
N1—Cu1—N2—C11	1.5 (3)	N3—C24—C16—C15	−1.2 (7)
C14—N3—C24—C16	1.2 (7)	C23—C24—C16—C15	−179.3 (4)
Cu1—N3—C24—C16	176.9 (3)	N3—C24—C16—C17	176.7 (4)
C14—N3—C24—C23	179.4 (4)	C23—C24—C16—C17	−1.4 (7)
Cu1—N3—C24—C23	−4.9 (5)	C13—C15—C16—C24	−0.2 (7)
O1—Cu1—N1—C1	83.4 (4)	C13—C15—C16—C17	−178.1 (5)
N2—Cu1—N1—C1	178.1 (4)	C19—C18—C17—C16	0.2 (7)
N3—Cu1—N1—C1	−9.1 (4)	C24—C16—C17—C18	0.6 (7)
N4—Cu1—N1—C1	−91.2 (4)	C15—C16—C17—C18	178.4 (5)
O1—Cu1—N1—C12	−95.9 (3)	C12—C4—C3—C2	−1.0 (7)
N2—Cu1—N1—C12	−1.1 (3)	C5—C4—C3—C2	179.2 (5)
N3—Cu1—N1—C12	171.7 (3)	C22—N4—C23—C19	2.7 (6)
N4—Cu1—N1—C12	89.6 (3)	Cu1—N4—C23—C19	180.0 (3)
O1—Cu1—N4—C22	94.6 (4)	C22—N4—C23—C24	−178.0 (4)
N2—Cu1—N4—C22	−12.9 (4)	Cu1—N4—C23—C24	−0.7 (5)
N3—Cu1—N4—C22	175.3 (4)	C20—C19—C23—N4	−1.8 (6)
N1—Cu1—N4—C22	−94.2 (4)	C18—C19—C23—N4	178.8 (4)
O1—Cu1—N4—C23	−82.1 (3)	C20—C19—C23—C24	178.9 (4)
N2—Cu1—N4—C23	170.3 (3)	C18—C19—C23—C24	−0.5 (6)
N3—Cu1—N4—C23	−1.5 (3)	N3—C24—C23—N4	3.7 (6)
N1—Cu1—N4—C23	89.0 (3)	C16—C24—C23—N4	−178.0 (4)
C10—N2—C11—C7	0.2 (7)	N3—C24—C23—C19	−176.9 (4)
Cu1—N2—C11—C7	178.5 (3)	C16—C24—C23—C19	1.3 (6)
C10—N2—C11—C12	−179.9 (4)	C4—C5—C6—C7	1.2 (8)
Cu1—N2—C11—C12	−1.6 (5)	C8—C7—C6—C5	179.5 (5)
C24—N3—C14—C13	0.4 (7)	C11—C7—C6—C5	−0.3 (7)
Cu1—N3—C14—C13	−174.7 (4)	C18—C19—C20—C21	179.3 (4)
C11—N2—C10—C9	1.5 (7)	C23—C19—C20—C21	−0.1 (7)
Cu1—N2—C10—C9	−176.5 (3)	C4—C3—C2—C1	1.5 (7)
C1—N1—C12—C4	−1.0 (6)	C23—N4—C22—C21	−1.7 (7)
Cu1—N1—C12—C4	178.3 (3)	Cu1—N4—C22—C21	−178.3 (4)
C1—N1—C12—C11	−178.7 (4)	C19—C20—C21—C22	1.0 (7)
Cu1—N1—C12—C11	0.7 (5)	N4—C22—C21—C20	−0.1 (8)
N2—C11—C12—N1	0.6 (6)	C12—N1—C1—C2	1.6 (6)
C7—C11—C12—N1	−179.6 (4)	Cu1—N1—C1—C2	−177.6 (3)
N2—C11—C12—C4	−177.2 (4)	C3—C2—C1—N1	−1.9 (7)
C7—C11—C12—C4	2.7 (6)	O4—S1—O1—Cu1	−145.3 (2)
N1—C12—C4—C3	0.7 (7)	O2—S1—O1—Cu1	−26.9 (3)
C11—C12—C4—C3	178.3 (4)	O3—S1—O1—Cu1	94.2 (3)
N1—C12—C4—C5	−179.4 (4)	N2—Cu1—O1—S1	72.8 (3)
C11—C12—C4—C5	−1.8 (6)	N3—Cu1—O1—S1	−111.3 (3)
N3—C14—C13—C15	−1.8 (8)	N4—Cu1—O1—S1	−33.9 (4)
C16—C15—C13—C14	1.6 (8)	N1—Cu1—O1—S1	154.6 (2)
C7—C8—C9—C10	0.3 (7)	O6—C26—C25—O5	71.3 (6)

### Hydrogen-bond geometry (Å, °)

**Table:**

*D*—H···*A*	*D*—H	H···*A*	*D*···*A*	*D*—H···*A*
O6—H6B···O4	0.82	1.93	2.751 (5)	176.
O5—H5B···O3	0.82	1.98	2.798 (5)	171.

## References

[bb1] Cui, J.-D., Zhong, K.-L. & Liu, Y.-Y. (2010). *Acta Cryst.* E**66**, m564. 10.1107/S1600536810014194 PMC2979185 21579046

[bb2] Flack, H. D. (1983). *Acta Cryst.* A**39**, 876–881.

[bb3] Jacobson, R. (1998). *REQAB.* Molecular Structure Corporation, The Woodlands, Texas, USA.

[bb4] Ni, C., Zhong, K.-L. & Cui, J.-D. (2010). *Acta Cryst.* E**66**, m746–m747. 10.1107/S1600536810020210 PMC3006809 21587684

[bb5] Rigaku (2007). *CrystalClear* Rigaku Corporation, Tokyo, Japan.

[bb6] Sheldrick, G. M. (2008). *Acta Cryst.* A**64**, 112–122. 10.1107/S0108767307043930 18156677

[bb7] Zhong, K.-L. (2010). *Acta Cryst.* E**66**, m247. 10.1107/S1600536810003478 PMC2983626 21580203

[bb8] Zhong, K.-L. (2011). *Z. Kristallogr. New Cryst. Struct.* **226**, 286–288.

[bb9] Zhong, K.-L. & Cui, J.-D. (2010). *Acta Cryst.* E**66**, m817–m818. 10.1107/S1600536810022518 PMC3006862 21587734

[bb10] Zhong, K.-L., Ni, C. & Wang, J.-M. (2009). *Acta Cryst.* E**65**, m911. 10.1107/S1600536809026269 PMC2977342 21583369

[bb11] Zhong, K.-L., Zhu, Y.-M. & Lu, W.-J. (2006). *Acta Cryst.* E**62**, m631–m633.

